# Integrating computational and chemical biology tools in the discovery of antiangiogenic small molecule ligands of FGF2 derived from endogenous inhibitors

**DOI:** 10.1038/srep23432

**Published:** 2016-03-22

**Authors:** Chiara Foglieni, Katiuscia Pagano, Marco Lessi, Antonella Bugatti, Elisabetta Moroni, Denise Pinessi, Andrea Resovi, Domenico Ribatti, Sabrina Bertini, Laura Ragona, Fabio Bellina, Marco Rusnati, Giorgio Colombo, Giulia Taraboletti

**Affiliations:** 1 Tumor Angiogenesis Unit, Department of Oncology, IRCCS-Istituto di Ricerche Farmacologiche Mario Negri, Bergamo, 24126 Italy; 2 Laboratorio NMR, Istituto per lo Studio delle Macromolecole, Consiglio Nazionale delle Ricerche, Milano, 20133 Italy; 3 Dipartimento di Chimica e Chimica Industriale, Università Di Pisa, Pisa, 56124, Italy; 4 Section of Experimental Oncology and Immunology, Department of Molecular and Translational Medicine, University of Brescia, Brescia, 25123 Italy; 5 Istituto di Chimica del Riconoscimento Molecolare, Consiglio Nazionale delle Ricerche, Milano, 20131 Italy; 6 Department of Basic Medical Sciences, Neurosciences and Sensory Organs, University of Bari Medical School, Bari, 70121 Italy, and National Cancer Institute “Giovanni Paolo II”, Bari, 70124 Italy; 7 Istituto di Ricerche Chimiche e Biochimiche G. Ronzoni, Milano 20133, Italy

## Abstract

The FGFs/FGFRs system is a recognized actionable target for therapeutic approaches aimed at inhibiting tumor growth, angiogenesis, metastasis, and resistance to therapy. We previously identified a non-peptidic compound (SM27) that retains the structural and functional properties of the FGF2-binding sequence of thrombospondin-1 (TSP-1), a major endogenous inhibitor of angiogenesis. Here we identified new small molecule inhibitors of FGF2 based on the initial lead. A similarity-based screening of small molecule libraries, followed by docking calculations and experimental studies, allowed selecting 7 bi-naphthalenic compounds that bound FGF2 inhibiting its binding to both heparan sulfate proteoglycans and FGFR-1. The compounds inhibit FGF2 activity in *in vitro* and *ex vivo* models of angiogenesis, with improved potency over SM27. Comparative analysis of the selected hits, complemented by NMR and biochemical analysis of 4 newly synthesized functionalized phenylamino-substituted naphthalenes, allowed identifying the minimal stereochemical requirements to improve the design of naphthalene sulfonates as FGF2 inhibitors.

Deregulation of FGFs (fibroblast growth factors) and their tyrosine kinase receptors (FGFRs) have a fundamental role in a variety of human cancers. Aberrant activation of this pathway, caused by gene amplification, chromosomal translocation, mutations, autocrine activation and impaired receptor down-regulation^1^,^2^ contributes to cancer progression by inducing tumor angiogenesis^3^,^4^,^5^. Moreover, FGFs directly promote tumor cell mitogenesis, survival, motility, invasion, epithelial-mesenchymal transition, and metastasis, and exert pleiotropic effects on the surrounding stroma^6^. In addition, FGFs/FGFRs have been reported to mediate tumor escape/resistance to VEGF-targeted therapies^7^ as well as resistance to targeted therapies such as Imatinib^8^.

The FGFs/FGFRs system is therefore a recognized actionable target to simultaneously affect angiogenesis, tumor cells and the stroma compartment. A rapidly expanding number of therapeutic compounds is being developed to target FGFs, their receptors, or downstream signaling, including tyrosine kinase receptor inhibitors, monoclonal antibodies, FGF traps, and ligands of the growth factors or their receptors^6^,^9^,^10^,^11^.

The activity of FGFs requires signaling triggered by a ternary complex formed by FGFs, cell surface heparan sulfate proteoglycans (HSPGs) and FGFRs^2^. The formation of this complex depends on the overall bioavailability of FGFs, regulated by their physical interactions with a variety of other molecules in the pericellular space. Among the FGF-binding extracellular molecules, we identified thrombospondin-1 (TSP-1) as a key regulatory ligand of FGF2^12^,^13^. We demonstrated that direct binding and sequestration of FGF2, through a sequence located in the type III repeats, is a mechanism of the antiangiogenic activity of TSP-1^14^, thus indicating that TSP-1 could represent a model for the development of new extracellular FGF2 inhibitors.

Structural analysis of the complex between FGF2 and the FGF2-binding domain of TSP-1 with the definition of pharmacophoric points led to the identification of new small molecule hits active in blocking FGF2 activity. The most active one, SM27 (NSC37204) binds specifically to FGF2, and inhibits FGF2-induced angiogenesis *in vitro* and *in vivo*^15^. Similarly to entire TSP-1, SM27 binds the heparin-binding site of FGF2. Binding of SM27 to the growth factor perturbs FGF2 dynamics in distant regions including the FGFR-1 binding site, thus preventing FGF2 binding to both HSPG and FGFR-1, with a final impairment of the ternary receptor complex formation required for FGF2 signaling^16^. Therefore, SM27 acts as a dual - direct and allosteric - inhibitor of the binding of FGF2 to its receptors, representing a viable lead for the development of novel cancer therapeutics.

In this work, we set out to expand the number of new small molecules targeting FGF2, by investigating the chemical space around SM27 and evaluating the activity of the resulting compounds with the perspective of evolving them into potential drug candidates. To this end, we first used molecular similarity criteria and docking calculations to screen the NCI database in search for molecules sharing with SM27 the structural and physico-chemical determinants necessary for binding to FGF2. Biophysical and chemical biology characterization of the compounds’ effects then highlighted promising activities for the newly discovered hits and allowed the definition of the structural requirements for FGF2 inhibition. Overall, our results show that the use of structural characterization of a starting complex, combined with chemo-informatics approaches can aptly be exploited for the discovery of new leads for further development into effective and more drug-like anticancer compounds.

## Results

### Identification of candidates

The Tanimoto coefficient based similarity search of novel leads that share with SM27 the same stereochemical determinants for activity returned 26 unique hits from the full NCI_2003 database, including mono- (SM.1 set) and bi-naphthalenes (SM.2 set). Given the peculiar chemical requirements for successful competition with heparin, and in order to obtain a diversity of hits sufficient to provide an initial indication of Structure Activity Relationships (SARs), we decided to use a rather permissive similarity criterion of 0.7.

**Figure 1:**
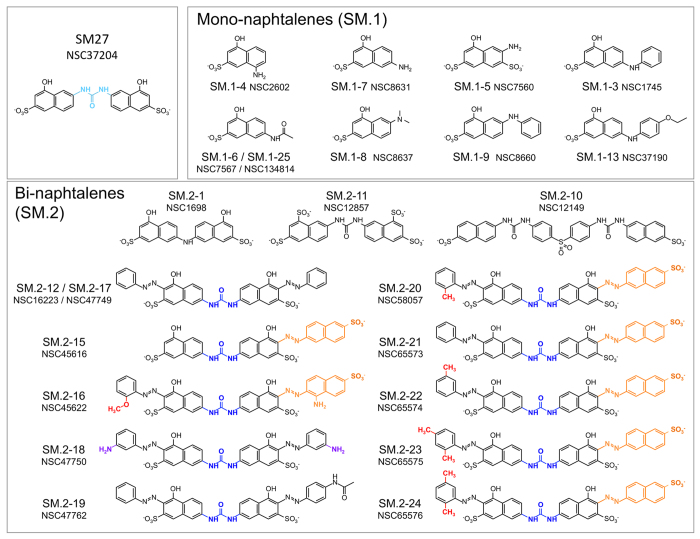
Structure of the lead compoud SM27 and the new identified hits, clustered according to structural features: mono-naphthalenes (SM.1 series) and bi-naphthalenes (SM.2 series). For the most active SM.2 compounds, the main stereochemical requirements for binding and activity are colored: ***blue***, urea bridge between the two napthalenes; ***orange***, sulfonate-decorated additional naphtyl-azo group; ***purple***, free amines on the two arylamine decorations in SM.2–18; ***red***, substituents on the aryl-azo group.

**Figure 2:**
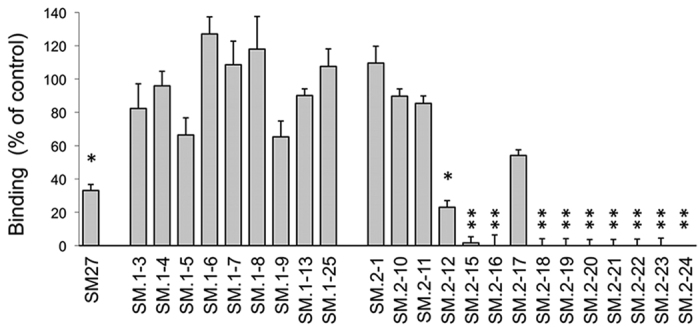
FGF2-binding property of the selected hits, analyzed with an indirect assay, as ability to compete with the TSP-1 fragment E123CaG1 (comprising the type III repeats domain) for FGF2 binding, as described in Methods. Binding of the biotinylated recombinant domain to FGF2 in the presence or absence of each molecule (5 μM). Data are the percentage of control binding, mean and SE of 3 experiments performed in triplicate. *p < 0.05 compared to control (ANOVA followed by Bonferroni test).

Of the identified hits, 23 (Fig. 1 and Suppl. Table S1), made available from the NCI (NIH), were subjected to experimental analysis of FGF2 binding. We used an indirect assay, based on the competition of the tested molecules with the recombinant TSP-1 type III domain for binding to FGF2, an approach previously employed to analyze the FGF2/SM27 binding^15^. A first screening of the 23 compounds, tested at the concentration of 5 μM, identified 10 hits that significantly prevented FGF2/TSP-1 binding (Fig. 2). Dose-response analysis (Suppl. Figure S1) was used to determine the IC50 value of competition (Table 1), and allowed identifying 7 hits (SM.2–16, SM.2–18, SM.2–19, SM.2–20, SM.2–22, SM.2–23, and SM.2–24), all bi-naphthalenes, that were more active (6–11 fold) than the initial query SM27, and were selected for further biochemical and functional characterization.

### Biochemical and functional analysis of bi-naphthalenic hits

Binding of 5 water-soluble compounds to FGF2 was further analyzed by surface plasmon resonance (SPR). All the compounds were able to bind FGF2 immobilized to a SPR sensorchip (Suppl. Figure S2). SM.2-20 bound FGF2 with an affinity (measured as dissociation constant, K_d_) comparable to the parental compound SM27 (318 and 337 nM, respectively). All the other compounds bound FGF2 with higher affinity (K_d_ values ranging between 39 and 125 nM) (Suppl. Table S2). In the context of biophysical characterization of compounds, we also tested the formation of aggregates in solution by representative compounds SM.2-16, SM.2-20 and SM.2-23, using Dynamic Light Scattering, which allows measuring nanoparticle size and polydispersion index (by Photocorrelation Spectroscopy) and surface charge (Zeta potential). For all the molecules, at concentrations between 0.03 and 1 mg/ml, the hydrodynamic radius ranged from 2.1 to 2.9 nm, with polydispersity index values between 0.5 and 0.6 (Table 2): no significant variation in the hydrodynamic radius was observed upon decreasing the concentrations, typically indicative of a non-aggregated state. Zeta potential values were strongly negative (indicative of molecule dissociation), ranging from −48 to −52 (Table 2). These findings therefore indicate that the solutions are fairly monodispersed, containing mainly single, charged molecules. This indicates that molecular aggregation/precipitation is not observable under the conditions used in the assays.

**Figure 3:**
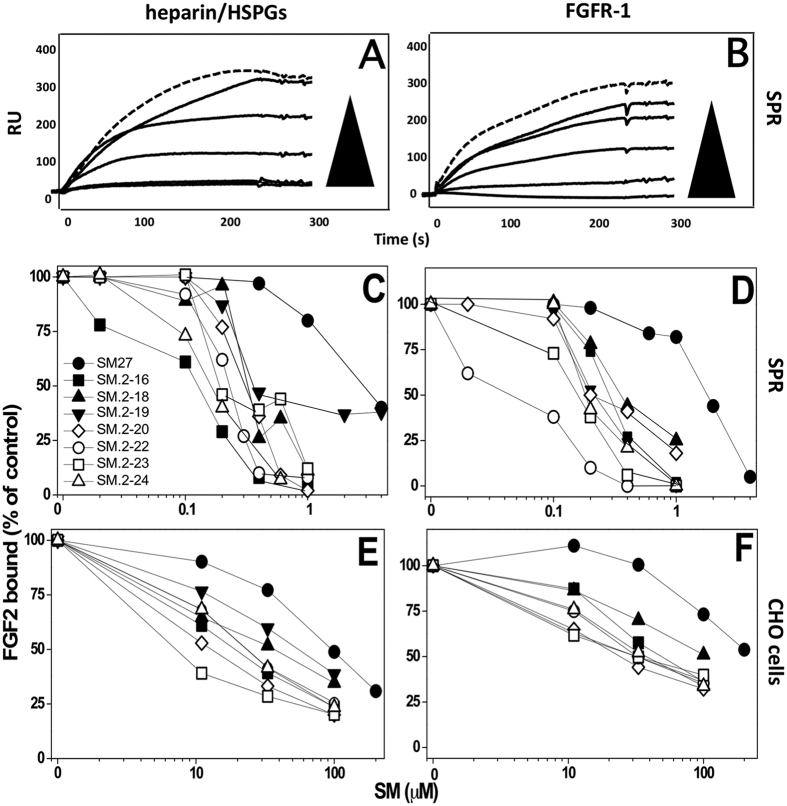
Inhibitory activity of the selected hits on the binding of FGF2 to heparin/HSPGs (**A,C,E**) or FGFR-1 (**B,D,F**). (**A,B)** Representative sensorgrams overlays derived by the injection of FGF2 (159 nM) in the absence (top hatched sensorgram) or in the presence of increasing concentrations of SM.2–22 on the heparin or FGFR-1 surfaces, respectively. (**C,D**) FGF2 (150 nM) was injected over heparin (**C**) or FGFR-1 (**D**) immobilized to a SPR sensorchip in the absence or in the presence of increasing concentrations of the indicated compounds and the amount of FGF2 bound to the surfaces in the different experimental conditions was measured. Each point is the mean of 3 experiments. (**E,F**) Binding of Eu-FGF2 (10 ng/ml) to CHO cell clones selectively expressing either HSPGs (**E**) or FGFR-1 (**F**) in the presence of increasing concentrations of the compounds. Each point is the mean of 2–3 experiments. Data are expressed as mean values of the percentage of bound FGF2 compared to control (in the absence of competing compounds).

We previously demonstrated that SM27 maintained the ability of TSP-1 to target the heparin recognition site of FGF2, preventing the FGF2 binding to heparin and HSPG^15^. The inhibitory effect of the selected hits on FGF2 interaction with heparin/HSPG was analyzed with two experimental approaches. First, SPR competition assays indicated that the newly identified bi-naphthalenes were active–and significantly more potent than SM27-in preventing FGF2 interaction with surface-immobilized heparin, a HSPG analog used in this binding assay and predictive of the FGF2/HSPGs interaction in cellular models^17^,^18^ (Fig. 3A,C). Next, a cell-based assay confirmed that the newly generated compounds were more potent than SM27 in inhibiting the binding of Eu-labelled FGF2 to CHO-K1 cells devoid of FGFR-1, in which the binding is thus entirely mediated by cell surface HSPGs (Fig. 3E).

SM27 acts with a dual mechanism of action to prevent the formation of the FGF2/FGFR-1/HSPGs ternary complex required for FGF2 activity. Besides blocking HSPG binding, the engagement of the heparin-binding site of FGF2 by SM27 also induces long-range dynamics perturbations of the FGF2 molecules at the FGF2/FGFR-1 interface regions, resulting in the inhibition of the FGF2 binding to FGFR-1^16^. We therefore investigated whether, besides their HSPGs antagonist capability, the newly selected hits maintained the parental compound’s property to prevent FGF2/FGFR-1 interaction. All the 7 new compounds were able to prevent the binding of FGF2 to FGFR-1 immobilized on a SPR sensorchip, with potencies that were always higher than SM27 (Fig. 3B,D). Similar findings were obtained when the capacity of the compounds to inhibit FGF2/FGFR-1 interaction was evaluated in the FGFR-1 over-expressing A745 CHO cells, deficient in HSPGs expression (Fig. 3F). Altogether these findings indicate that the selected hits retain the ability of the parental compound SM27 to act by the dual (anti-HSPGs and anti-FGFR-1) mechanism, but with significantly increased potency compared to the original query.

**Figure 4:**
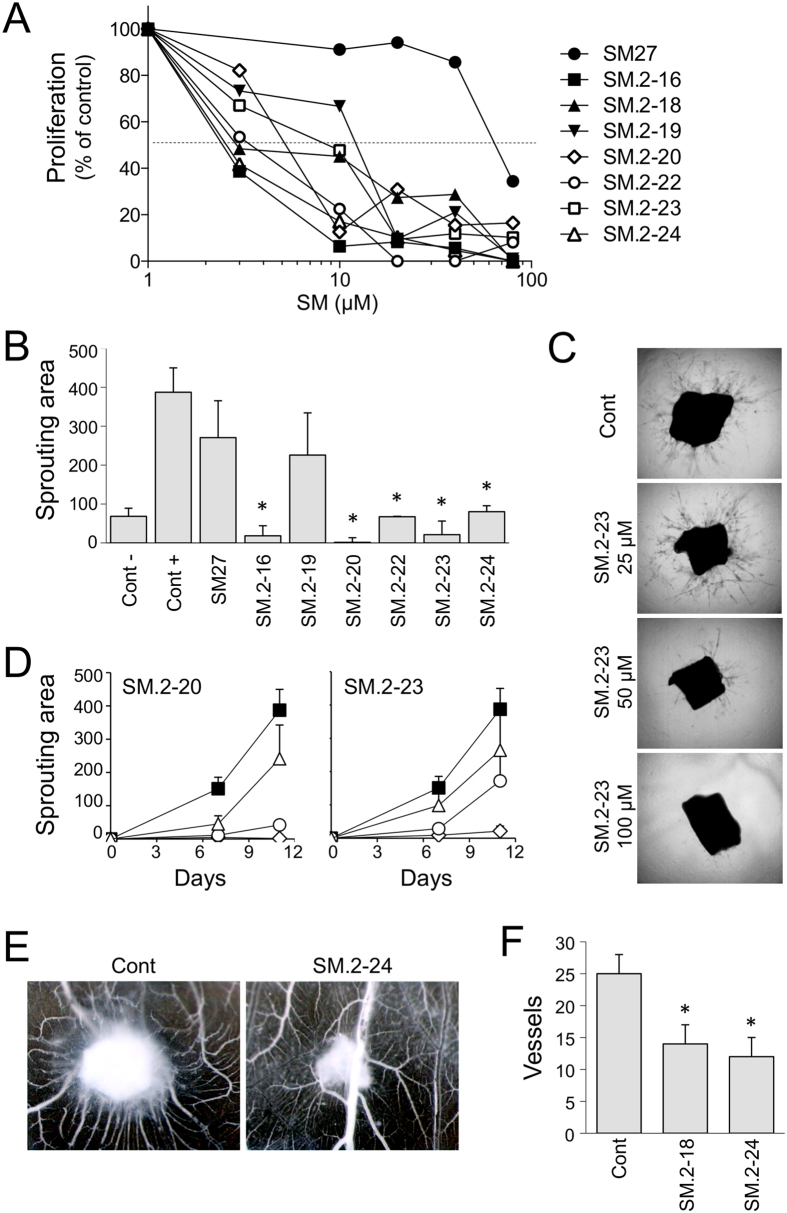
Biological activity of the selected hits. (**A**) Endothelial cell proliferation. BAEC were exposed to FGF2 (5 ng/ml) with increasing concentrations of molecules (3–80 μM). After 72 h, cells were stained and proliferation measured as absorbance. Data are the percentage of control proliferation (in absence of molecules), mean of value from 2 experiments performed in triplicate. (**B–D**) Aortic ring assay. Sections of murine aortas were embedded in Matrigel, in the presence of FGF2 (30 ng/ml) and the indicated small molecule. The formation of capillary structures sprouting from the rings was analyzed after 7 and 11 days as described in Methods, and the angiogenic response expressed as area covered by the sprouting structures (arbitrary units, mean and SE, n ≥ 6). (**B**) Antiangiogenic activity of the small molecules (100 μM). (**C**) Examples of time-dependent and dose-dependent effect of two compounds (SM.2–20 and SM.2–23), tested at 100 (diamond), 50 (circle) and 25 μM (triangle) compared to control (black squares). (**D**) Representative pictures of sprouting from control and SM.2–23 treated aortic sections. Original magnification, 20x. (**E,F**) Chorioallantoic membrane assay. FGF2 (200 ng) was administered in the absence or presence of the indicated compound (0.5 μg) on day 8 (n = 10). (**E**) Angiogenic response is evaluated 4 days later, and expressed as number of vessels entering the sponge (mean and SD). (**F**) Representative pictures are shown. Original magnification, 50x.

All the new hits inhibited FGF2-induced endothelial cell proliferation with potencies that were always significantly higher than SM27 (Fig. 4A). Further analyses were conducted on selected hits, chosen on the basis of availability and solubility properties. SM.2–16, SM.2–20, SM.2–22, SM.2–23 and SM.2–24, though not SM.2–19, significantly inhibited FGF2-induced vessels sprouting in the *ex vivo* model of murine aortic rings in a 3D Matrigel support (Fig. 4B–D). SM.2–18 and SM.2–24 were also tested and found to be active in inhibiting FGF2-induced angiogenesis in the CAM assay (Fig. 4E,F).

These findings confirm that second-generation bi-naphthalenic small molecules, based on the FGF2 binding sequence of TSP-1, bind and sequester FGF2, and inhibit its angiogenic activity with increased potency over the original lead SM27.

### Docking analysis of bi-naphthalenic hits

**Figure 5:**
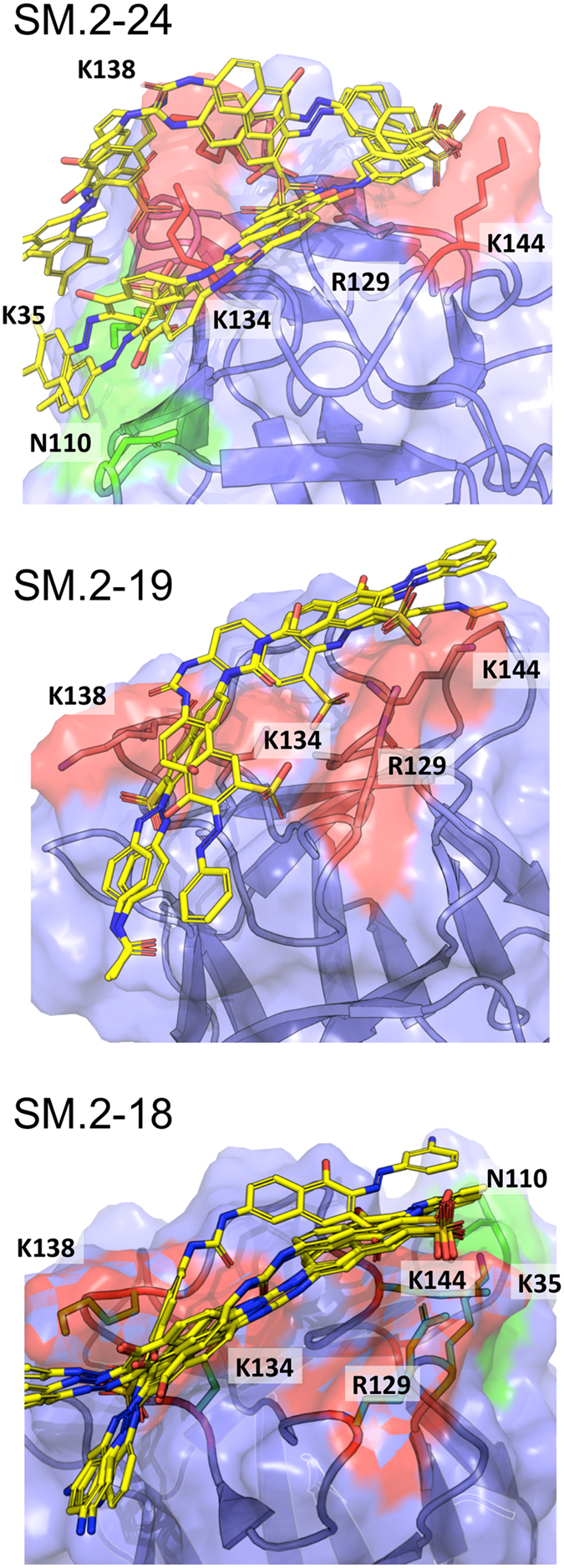
The best poses of SM.2–24, SM.2–19 and SM.2–18, resulting from the ensemble docking of the ligands into the heparin-binding pocket of FGF2. In green: area of additional interactions compared to SM.2–19.

The interaction of the novel hits with FGF2 was analyzed by docking studies. The molecules were able to engage the heparin-binding site of FGF2, as expected given the above findings and their similarity to SM27 (Fig. 5). Considering that the small molecules have to engage a superficial region of the protein endowed with conformational flexibility, we selected an ensemble approach to characterize FGF2-ligand interactions. Indeed, in contrast to ligands binding to classical rigid targets such as enzyme active sites that usually results in a dominant bound structure, small molecule targeting of large and dynamic superficial regions may be better described as an ensemble of ligand structures around a diverse set of protein conformations^19^,^20^,^21^. We therefore analyzed different poses for each ligand on the binding surface of FGF2 (Fig. 5). All molecules interact with Arg129 and Lys144, consistent with previous MD and NMR results on SM27^16^. The principal docking poses of SM.2–24, representative of the family of the most active compounds, including SM.2–16, SM.2–20, SM.2–22, and SM.2–23 (see above and Fig. 4), indicate that the main factors for improved IC50 of this new set of leads are the extension of the hydrophobic/aromatic contact surfaces with the protein, and the formation of charge-charge interactions between the sulfonate on the additional naphtyl-azo group on the naphthalene ring (orange in Fig. 1). Specifically, the aromatic extension optimally packs on the surface of FGF2 (Fig. 5) and allows the additional SO3 group to point toward Lys134 and Lys138, establishing additional stabilizing interactions. These interactions are observed in all the main poses of SM.2–24 bound to FGF2 (Fig. 5) as well as in the case of Sm.2–16, SM.2–20, SM.2–22, and SM.2–23. Compound SM.2–19, which shows the lowest activity (see above and Fig. 4) lacks these additional interactions: structural analysis indeed indicates that no stable additional charge-charge contact can be established with Lys134 and Lys138 (Fig. 5) and the packing with the surface is significantly diminished. The structural data also provide a possible molecular rationale for the observed lower inhibitory activities of SM.2–19 compared to the SM.2–18. In SM.2–18 the additional free amino group on each of the two arylamine decorations (purple in Fig. 1) allows the formation of stabilizing H-bonding interactions with Asn110 and Lys35 on one side of the ligand and with the side chain of Lys144 on the other (Fig. 5).

### Activity and NMR analysis of new anilino-naphthalenic compounds

To further characterize the molecular interactions and to gain further insights into the minimal stereochemical requirements for FGF2 recognition, 4 functionalized phenylamino-substituted naphthalenes were synthesized and used as elemental chemical probes for biochemical and biophysical analysis.

**Figure 6:**
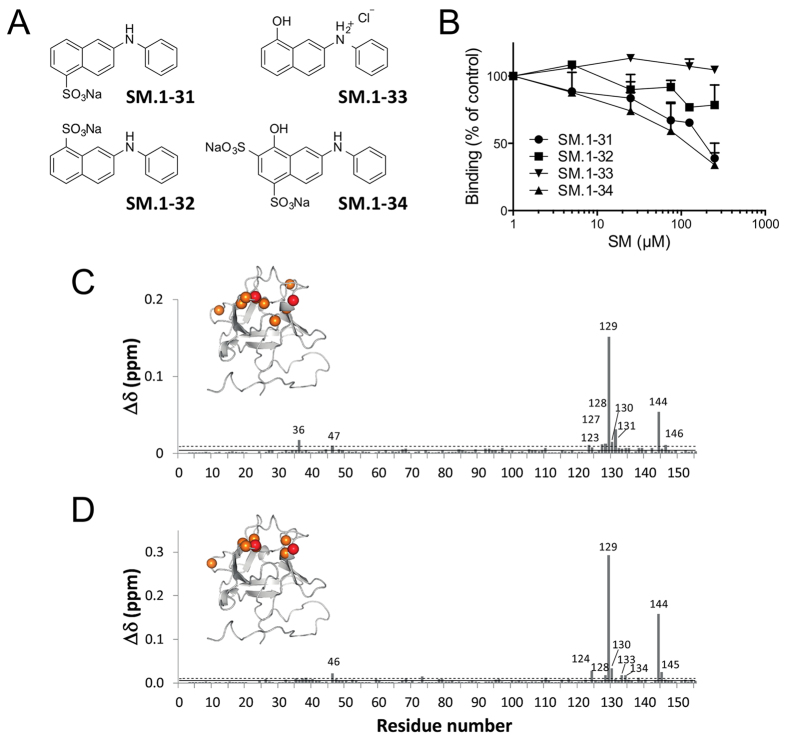
(**A**) Structure of the 4 phenylamino-substituted naphthalenes synthesized and analyzed in the present study. (**B**) Antiproliferative activity of the four anilino-naphthalenes was analyzed as in Fig. 4A. Data are the percentage of control proliferation (in absence of molecules). (**C**) Graphical representation of the combined ^1^HN and ^15^N FGF2 chemical shift perturbation determined for the various residues, according to^36^ 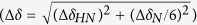, following the addition of phenylamino-substituted naphthalenes. **C)** Chemical shift perturbation (CSP) induced by SM.1–34 addition in SM:FGF2 1:1 stoichiometric ratio **D)** CSP induced by SM.1–31 addition in SM:FGF2 1:3 stoichiometric ratio. The continuous and dashed lines represent the average and the average plus a standard deviation (SD) values, respectively. Residues affected by CSP > (<CSP>) + 1SD are mapped on the FGF2 structures as orange spheres. Residues Arg129 and Lys144, showing the highest CSP, are shown as red spheres.

The four compounds (Fig. 6A) presented different ability to bind FGF2, from the inactive SM.1–33 to SM.1–31 and SM.1–34, which were the most active in the competition assay (Fig. 6B).

In order to investigate whether the functionalized phenylamino-substituted naphthalenes maintained the ability of TSP-1 and SM27 to target the heparin interaction site of FGF2^16^,^21^, interaction studies between SM.1–31 or SM.1–34 (showing the highest activity) and FGF2 were performed by NMR. Each SM was titrated into a solution of ^15^N-enriched FGF2 protein and a series of 2D ^1^H^15^N-HSQC experiments were recorded to follow the protein changes induced by the ligand. The titration progress was suggestive of an intermediate to fast exchange phenomenon, indicative of affinities in the high micromolar range. The chemical shift perturbation (CSP) analysis of the FGF2 resonances (Fig. 6C,D) clearly indicated that the two molecules share a common binding site, located in the long FGF2 b10-b12 loop, which is part of the reported heparin binding site. Residues showing the highest perturbations are Arg129 and Lys144, as previously observed for SM27, pointing to similar interactions that likely involve inhibitors SO3 groups. Notably, a difference in potency was observed between the isomers SM.1–31 and SM.1–32, which differ in the position of the sulfonate group.

## Discussion

The great potential of FGF2 inhibitors as antineoplastic therapeutics lies in the fact that a number of tumor types are intrinsically dependent on the FGF/FGFR signaling, that induces angiogenesis, exerts pleiotropic effects on both the tumor cells and the surrounding stroma and mediates resistance to antiangiogenc and targeted therapies^1^,^2^,^6^,^8^.

Our approach to identify FGF2 inhibitors was based on the hypothesis that endogenous inhibitors of this growth factor could serve as models for the rational design of new inhibitors to be used in the treatment of cancer^22^. Following this approach, we previously identified a novel FGF2 inhibitor, SM27, mimetic of an antiangiogenic sequence in the type III domain of the endogenous angiogenesis inhibitor TSP-1^15^,^23^. SM27 had a dual effect, inhibiting FGF2 binding to both HSPG (direct competition) and FGFR-1 (allosteric inhibition)^16^, hence emerging as a promising lead for the development of derivatives targeting the FGF2/HSPG/FGFR ternary complex required for signaling.

The development of molecularly targeted small molecule drugs from active leads typically involves iterative cycles of identification/design flanked by molecular and biological evaluation, to progressively improve potency, selectivity and pharmacological properties. Hence we followed this procedure to select a set of second-generation FGF2 targeting compounds based on the original lead SM27, with an improved capacity to interfere with FGF2 protein-protein interactions. The approach we used and the identified derivatives provide a convenient framework to gain relevant insights into the determinants for FGF2 recognition. It is worth noting here that some critical aspects of second-generation compounds, (i.e. the presence of hydrophobic and charged groups and the potential reactivity of the azo-spacer), require further development and medicinal chemistry optimization. However, it must be considered that as the leads need not be cell-permeable, we allowed for the inclusion of additional charged and hydrogen bonding functionalities on the identified scaffolds. Moreover, biophysical, structural and computational characterizations of binding of second-generation leads to isolated FGF2 prove that there is direct interaction with the target and that this interaction is significantly modulated by the stereoelectronic properties of the ligands and by the conformational cross-talk between the binding partners. In this framework, the SARs of the new angiogenic inhibitors can be dissected and the resulting most relevant functionalities used to define pharmacophoric points for further medicinal chemistry based development of the leads, as discussed below.

The chemo-informatic search resulted in two main classes of mono- and bi-naphthalenic compounds. Ten hits were able to interact with FGF2, 7 of which - all bi-naphthalenic - were more active than the original lead SM27 and were hence selected for further analysis. Similar to SM27, all these compounds were able to engage the heparin-binding site of FGF2, as observed from docking studies, and interfered with FGF2 binding to both HSPG and FGFR-1 in cell-free and cell culture models. Altogether these findings point to a direct inhibition of FGF2/HSPG binding accompanied by allosteric effects on the FGF2/FGFR-1 interaction, in agreement with the what previously described for SM27^16^. In line with these findings, heparin and heparin-like molecules have been described to affect FGF conformation and backbone dynamics, with different effects depending on the polysaccharide properties and the FGF subfamily^24^,^25^,^26^. In particular, the naphthalene compound 5-amino-2-naphthalenesulfonate, has been reported to inhibit both FGF1 and FGF2^27^, a property whose *pro* and *cons* are still debated in view of the redundancy and promiscuity of the FGF/FGFR system in cancer. Along this line, the possibility that also the different hits here identified might show a multitarget binding capacity for different members of the FGF family rather than a FGF2 selectivity warrants further investigation.

A comparative analysis of the experimental data obtained with the selected hits as well as the 4 functionalized phenylamino-substituted naphthalenes reported here allows drawing some consideration on the stereochemical requirements to improve the design of naphthalene sulfonates as FGF2 inhibitors. Analysis of the docking models of a selected subset of active compounds (SM.2–16, SM.2–18, SM.2–20, SM.2–22, SM.2–23 and SM.2–24) in the light of the experimental results on their biological functions, highlights relevant features correlating their structures and activities: the urea bridge (blue in Fig. 1) appears to be important to ensure the optimal distance between the SO3 groups on the napthalenic derivatives directly linked to the bridge, without apparently establishing specific electrostatic interactions, as indicated by the different aromatic carbon atoms involved in the C-N bonds with the two nitrogen atoms of the urea unit. In fact, in SM-27 the urea bridge is in a 1, 3 relative position with the phenolic OH group, while in active SM.2 compounds the same functional unit is in a 1, 4 positional relationship with the same OH group. The relevance of the sulfonate group on the naphthalene rings is confirmed using 4 phenylamino-substituted naphthalenes as chemical tools. Indeed a significant loss of activity is observed in SM.1–33, missing the SO3 group at its C-6 and decorated with a hydroxyl group at its C-8. Nonetheless, the hydroxyl group in meta to a sulphonyl group is a requirement, since the activity of SM27 is lost in SM.2–11, where the two phenolic OH groups are substituted by SO3 groups. The position of the SO3 decoration also affects the antiproliferative activity, since an increased activity is observed in SM.1–31, with the group located in 1, 4 relative position in respect to the amino group, when compared to SM.1–32, in which the same groups are linked to the naphthalenic core in a 1, 3 fashion.

The most active compounds are characterized by the presence of an aryl-azo group on the naphthalene ring between the OH and SO3 functionalities. Specific substituents on this ring relevantly contribute to the interaction, as shown by the reduced activity of SM.2–12/SM.2–17, lacking charged substituents on the phenyl-azo groups. Based on experimental functional data and on the structures emerging from docking calculations, we suggest that the substituents may carry out their effects through a combination of factors: first, the hydrophobic nature of the additional aromatic moieties can be beneficial in establishing further hydrophobic interactions with FGF2 surface. Second, substitutions on the aryl-azo function determine a modulation of the activity of the ligand, which can be rationalized as follows: i) in the case of a ortho-substitution with a methyl substituent, steric hindrance induces a significant bending in the whole molecule, which turns out to be beneficial for pointing the two SO3- on the naphthalene rings linked to the urea bridge to optimally establish electrostatic interactions with the FGF2 positively charged residues. Moreover, we speculate that the bending induced by the substitution could pre-organize the ligand in solution, favoring conformations that are similar to the bound ones, selecting preferred conformations for binding and minimizing of the entropic costs associated to binding; ii) in the case of the presence on the aza-aromatic substituents of sulfonate or amino groups, additional charge or hydrogen bonding interactions with the surface can be formed. The latter can clearly increase the affinity of the leads for the target, which expectedly reverberates in higher activities. This set of observations can be used to advance the understanding of the stereoelectronic determinants of FGF2-binding into the design of novel compounds with improved drug-like profiles. In this context, the azo-group should be considered as a spacer to allow decoration of the main pharmacophoric group (the SM27-like core) with functionalities that allow extending interactions into previously unexplored pockets. Therefore, as the aza-group has the potential to exert off targets effects (possibly accounting for the lack of a strict correlation between Kd values biophysically determined in binary binding assays and the inhibitory activities observed in cells), our SAR analysis indicates that such reactive spacer may aptly be substituted by non-reactive linkers allowing the same distance between groups, with a marked improvement in the compound specificity. Moreover, we suggest that the pharmacologic potential of the compounds may be improved by the replacement of naphtyl-rings with substructural motifs that preserve the optimal orientations of charged and h-bonding functionalities while minimizing non-specific hydrophobic effects.

Polysulfonated macromolecules such as suramin (a polysulfonated binaphthyl urea) and suradista (a bi-naphthalene sulfonic distamycin-A derivative) have been developed as inhibitors of angiogenic factors. Searching for the minimal active substitutes of suramin and suradista, Giménez-Gallego and coworkers identified two naphthalene sulfonates as antiangiogenic candidates, naphthalene-trisulfonate^28^ and 5-amino-2-naphthalenesulfonate (ANSA)^27^. Similarly, our search for molecules structurally similar to SM27 identified several amino-naphthalene sulfonates (SM.1–4 to SM.1–8). These compounds were however less potent than SM27, suggesting that mono-naphthalenic small molecules do not fully recapitulate the activity of the original hit and of larger macromolecules such as TSP-1. Conversely, the 7 bi-naphthalenic compounds selected in this study present a much higher activity in terms of inhibition of FGF2 mitogenic activity (IC50 ranging from 3.4 to 13.8 μM) compared to 5-amino-2-naphthalenesulfonate (IC50 265 μM)^27^, hence emerging as effective step forward toward the development of novel potent FGF2 antagonists. This possibility is supported by the observation that the hits were more potent than SM27 in inhibiting pro-angiogenic activities of FGF2 in more complex models of angiogenesis.

In conclusion we have identified a set of second-generation, low molecular weight FGF2 inhibitors, characterized by the prominent ability to affect the FGF2/HSPG/FGFR complex through a dual (direct and allosteric) mechanism, and have improved affinities compared to the original hit. The analysis of their efficacy and safety in preclinical tumor models will allow determining their actual potential as antineoplastic compounds, while assessing the possible need for further derivation of new leads form these structures.

## Methods

### Inhibitors

SM.1–31, SM.1–32, SM.1–33, SM.1–34 were synthesized and characterized by ^1^H and ^13^C NMR as described in details in the Supplementary Methods, and Suppl. Figures S3 and S4. The other compounds (Fig. 1 and Suppl. Table S1) were provided by the Developmental Therapeutics Program (DTP), division of Cancer Treatment and Diagnosis, NCI, NIH (USA).

### Reagents

Human recombinant FGF2 (R&D Systems, Minneapolis, MN) was obtained through the NCI-Biological Resources Branch (Frederick, MD). The uniformly ^15^N-labelled 154-residue form of Glu^3^,^5^ Ser^78,96^ human recombinant FGF2 was produced and purified by ASLA (Riga, Latvia) according to the protocol previously optimized^29^,^30^. The human recombinant TSP-1 fragment comprising the FGF2 binding region (E123CaG1)^14^,^15^,^31^ was kindly provided by D.F. Mosher (Madison, WI).

### Computational analysis: similarity based identification of new leads

The identification of novel leads started with the screening of the whole NCI database in search for molecules sharing with SM27 the functionalities necessary for binding FGF2. To this end, a Tanimoto-similarity coefficient^32^ of 0.7 to SM27 was used, as implemented in the Discovery Studio suite of programs. The value of 0.7 was empirically chosen to return a reasonable number of testable hits from the NCI library, containing around 300000 compounds: 41 molecules were initially selected. After close examination of the structures 15 were excluded as redundant (representing different conformers, tautomeric states, etc.) and 26 unique molecules were identified and retained for further computational and experimental analysis.

### Molecular docking of selected compounds on FGF2

The initial target for docking of the selected compounds was FGF2 structure 1FQ9.pdb, used for previous studies of SM27 binding^16^. All docking calculations were carried out using a flexible receptor and flexible ligand approach, as implemented in the program Glide (version 5.8 Schrödinger, LLC, New York, NY, 2012). Docking calculations were performed in Standard Precision mode (SP) with standard OPLS-AA (2001) force field, non-planar conformations of amide bonds were penalized, Van der Waals radii were scaled by 0.80 and the partial charge cut off was fixed to 0.15. No further modifications were applied to the default settings.

### Labeling of proteins

The recombinant TSP-1 domain was biotinylated with biotinamidocaproate-N-hydroxysulfo-succinimide ester (Sigma, St. Louis, MO), and isolated by chromatography on Micro Bio-Spin columns (Bio-Rad Lab, Milan, Italy) as described^14^,^15^. FGF2 was labeled with the lanthanide Europium using Europium-labeling reagent (PerkinElmer) and purified on Heparin Sepharose as described^15^.

### Binding of the TSP-1 domain to FGF2

DELFIA^®^ Microtitration plates and reagents were from PerkinElmer. Plates were coated overnight at 4 °C with FGF2 (0.1 μg in 40 μl /well PBS). After washing, non-specific binding sites were saturated by a 30 minute incubation with PBS 1% BSA. The biotin-labeled recombinant TSP-1 fragment (10 nM) was added in 40 μl PBS 1% BSA with or without the indicated concentration of small molecules, and incubated for 3 h at room temperature. The plates were washed with PBS 0.1% BSA and incubated for 1 h with 100 μl/well Eu-Labeled Streptavidin 1:1000 at room temperature. After washing, plates were incubated with DELFIA^®^ Enhancement Solution. Time resolved fluorescence was measured using a Victor^3^ multilabel plate reader (PerkinElmer).

### Surface plasmon resonance (SPR) analysis

SPR measurements were performed on a BIAcore X100 instrument (GE-Healthcare, WI). FGF2 was immobilized on a CM5 sensorchip (GE-Healthcare) as described^13^, allowing the immobilization of 7,365 resonance units (RU) (410 fmol/mm^2^ of FGF-2). Similar results were obtained for the immobilization of bovine serum albumin (BSA), used as a negative control and for blank subtraction.

FGFR-1 was immobilized on a CMD50L sensorchip (Xantec Bioanalytics, Dusseldorf, Germany) as described^16^, allowing the immobilization of 3,800 RU (42.0 fmol/mm^2^ of FGFR-1). Similar results were obtained for the immobilization of FGF-unrelated noggin protein, here used for blank subtraction. To immobilize heparin onto the SPR chip, a CMD50L sensorchip was activated and coated with streptavidin. Heparin was biotinylated and immobilized onto the streptavidin-coated sensorchip, allowing the immobilization of 80 RU (6.0 fmol/mm2 of heparin)^16^. A streptavidin-coated sensorchip was used for blank subtraction.

To analyze their direct binding to sensorchip-immobilized FGF2, the compounds were resuspended in 10 mM HEPES, 150 mM NaCl, 3.4 mM EDTA, 0.005% surfactant P20, pH 7.4 (HBS-EP) at increasing concentrations (from 62.5 to 1,000 nM), injected for 5 min (to allow their association to FGF2) and washed until dissociation was observed. SPR analysis of higher concentrations of compounds were not taken in consideration because of abnormal RU signals, possibly due to their tendency to form aggregates at high concentrations.

For competition experiments, FGF2 (150 nM) in the presence of increasing concentrations of the compounds (from 10 to 5,000 nM) in HBS-EP was injected over the heparin or FGFR-1 surfaces for 5 min (to allow the association of the growth factor with the receptors) and washed until dissociation was observed. In both the experimental conditions, the sensorchips were regenerated after every run by injection of glycine 10 mM pH 1.5.

### Photon correlation spectroscopy and Zeta Potential analysis

The Photon correlation spectroscopy (PCS) and the Zeta Potential (Zp) values were measured using the Zetasizer Nano ZS (Malvern, Worcestshire, UK), as detailed in Supplementary Material. Data were analyzed by Zetasizer software, version 7.11 (Malvern, Worcestshire, UK).

### Cell cultures

The isogenic cell model expressing either HSPG or FGFR-1 consisted of wild-type CHO-K1 cells provided by J.D. Esko (La Jolla, CA)^33^, expressing HSPG (but not FGFR-1), and the derived HSPG-deficient A745 CHO cell mutants (by J.D. Esko), transfected with the IIIc variant of murine FGFR-1 cDNA^34^. Cells were grown in Ham’s F-12 with 10% FCS. Bovine aortic endothelial cells (BAEC) were cultured in DMEM with 10% FCS.

### Binding of FGF2 to cells

Subconfluent cultures of HSPGs-expressing CHO-K1 or FGFR-1-expressing A745 CHO cells in 96-well plates were incubated for 30 min at 4 °C in serum-free DMEM with 0.15% gelatin and 25 mM HEPES (DMEM-gelatin). The medium was then replaced with cold DMEM-gelatin containing Eu-labeled FGF2 (10 ng/ml) with or without the indicated concentration of small molecules. The plates were incubated for 2 h at 4 °C. Wells were washed with cold DMEM-gelatin to remove unbound FGF2, and the amount of total bound FGF2 was detected by adding DELFIA^®^ Enhancement Solution and measuring time resolved fluorescence as described^16^.

### Endothelial cell proliferation assay

BAEC (2500 cells/well) were plated in 96-well plates in DMEM 1.5% FCS. After 24 h, the medium was substituted with DMEM 0.5% FCS, with or without FGF2 (5 ng/ml) and the indicated concentration of small molecules, and incubated for 72 h. Plates were then stained with crystal violet or MTS and analyzed as described^15^.

### Aortic rings assay

C57BL/6 mice (Harlan, Correzzana, Italy) were used. Mice were housed under specific pathogen-free conditions. All the procedures were conducted in conformity with: Italian Law (D.lgs 26/2014; Authorisation n.19/2008-A issued March 6, 2008 by Ministry of Health); Mario Negri Institutional Regulations and Policies providing internal authorization for persons conducting animal experiments (Quality Management System Certificate – UNI EN ISO 9001:2008 – Reg. N° 6121); the NIH Guide for the Care and Use of Laboratory Animals (2011 edition) and EU directives and guidelines (EEC Council Directive 2010/63/UE); Statement of Compliance with the Public Health Service, expiring in 2019 (Animal Welfare Assurance #A5023-01). Experimental procedures have been authorized by the Ministry of Health.

Thoracic aortas were removed from mice, cleaned of perivascular adipose tissue and cut into 1 mm segments. Matrigel (75 μl, BD Biosciences) was added to 96 well plates, and allowed to gellify at 37 °C for 30 min. Aortic sections were added to the wells, and covered with a second layer of Matrigel and DMEM 5% FCS containing FGF2 (30 ng/ml) and the indicated concentration of the compounds. The formation of capillary structures sprouting from the rings was analyzed after 4, 7 and 11 days with an inverted-phase contrast microscope (IX70; Olympus). Images were analyzed with ImageJ 1.48, and the angiogenic response expressed as area covered by the sprouting structures (arbitrary units), substracted the area of the aortic section.

### Chorioallantoic membrane (CAM) assay

Fertilized White Leghorn chicken eggs were incubated at 37 °C at constant humidity. On day 3, a square window was opened in the shell, 2–3 ml of albumen was removed to allow detachment of the developing CAM, and the window was sealed with a glass. On day 8, 1 mm^3^ sterilized gelatin sponges (Gelfoam Upjohn, Kalamazoo, MI) were placed on the CAM as described^35^. Sponges were loaded with: PBS or FGF2 (200 ng) with or without the indicated molecule (0.5 μg). At day 12, CAMs were photographed *in ovo* under a stereomicroscope (Olympus), and blood vessels entering the sponge within the focal plane of the CAM were counted by two observers in double blind.

### NMR characterization of FGF2/phenylamino-substituted naphthalenes interactions

NMR samples contained 0.57 mM ^15^N-labeled FGF2, dissolved in a buffer containing 50 mM potassium phosphate, 2 mM NaN3, and 10 mM deuterated DTT in 90% H2O/10% D2O, pH 5.5. For the identification of the binding region, SM.1–34 and SM.1–31 were incrementally added to protein solutions up to SM:FGF2 stoichiometric ratio of 3. NMR data were collected on a Bruker DMX 500 MHz spectrometer at 25 °C. 2D ^1^H-^15^N HSQC spectra were recorded with a sweep width of 13 ppm (^1^H) and 40 ppm (^15^N). 1k × 128 data points were used in proton and nitrogen dimensions, respectively. Data, acquired and processed using Topspin (Bruker Biospin), were apodized with a squared sinebell shifted by 90° and polynomial baseline correction. The analysis was performed by means of Sparky (T.D. Goddard and D.G. Kneller, SPARKY3, University of California, San Francisco).

### Statistical analysis

Statistical significance of differences was assessed with ANOVA followed by Dunnett or Bonferroni post-test, using Prism 6 software (GraphPad, La Jolla, CA).

## Additional Information

**How to cite this article**: Foglieni, C. *et al.* Integrating computational and chemical biology tools in the discovery of antiangiogenic small molecule ligands of FGF2 derived from endogenous inhibitors. *Sci. Rep.* **6**, 23432; doi: 10.1038/srep23432 (2016).

## Supplementary Material

Supplementary Information

## Figures and Tables

**Table 1:** FGF2 binding ability of the selected hits (competition assay).

Group	Molecule	IC_50_ (μM)
*Initial query*	*SM27*	*3.90* ± *0.23*
Bi-naphthalene	SM.2–16	0.59 ± 0.09
Bi-naphthalene	SM.2–18	0.61 ± 0.06
Bi-naphthalene	SM.2–19	0.35 ± 0.03
Bi-naphthalene	SM.2–20	0.50 ± 0.11
Bi-naphthalene	SM.2–22	0.42 ± 0.09
Bi-naphthalene	SM.2–23	0.53 ± 0.11
Bi-naphthalene	SM.2–24	0.46 ± 0.10

The ability of the selected hits to compete for the binding of the recombinant TSP-1 domain to FGF2 was analyzed as an indirect measure of their ability to bind FGF2, as previously described^15^. Data are the IC_50_ (in μM), mean ± SE of data from 3 experiments.

**Table 2:** Dynamic Light Scattering analysis of the formation of aggregates by representative compounds in solution.

Compound	Concentration (mg/ml)	HydrodynamicRadius (nm)	PdI	Zeta potential
SM.2–16	1	2.283	0.695	−48.0
	0.5	2.265	0.533	
	0.25	2.511	0.663	
	0.125	2.149	0.452	
	0.0625	2.364	0.742	
	0.03125	2.140	0.495	
SM.2–20	1	2.878	0.595	−50.2
	0.5	2.571	0.518	
	0.25	2.480	0.789	
	0.125	2.172	0.635	
	0.0625	2.159	0.658	
	0.03125	2.186	0.523	
SM.2–23	1	2.877	0.545	−52.2
	0.5	2.597	0.539	
	0.25	2.951	0.59	
	0.125	2.765	0.557	
	0.0625	2.370	0.677	
	0.03125	n.d.	n.d.	

Nanoparticle size and polydispersion index (by Photocorrelation Spectroscopy) and surface charge (Zeta potential) were measured as an index of aggregation of the three compounds in solution, tested at the indicated concentration.
