# Supplementary material for: Integrating computational and chemical biology tools in the discovery of antiangiogenic small molecule ligands of FGF2 derived from endogenous inhibitors
Source: Sci Rep. 2016 Mar 22;6:23432. doi: 10.1038/srep23432 (PMC4802308; doi:10.1038/srep23432)
Supplement: Supplementary Information [file srep23432-s1.pdf]

Integrating computational and chemical biology tools in the discovery of antiangiogenic small molecule ligands of FGF2 derived from endogenous inhibitors

Foglieni C, Pagano K, Lessi M, Bugatti A, Moroni E, Pinessi P, Resovi A, Ribatti D, Bertini S, Ragona L, Bellina F, Rusnati M, Colombo G, Taraboletti G

SUPPLEMENTARY MATERIAL

SUPPLEMENTARY METHODS

Inhibitors. Fluka precoated 60 F254 aluminium silica gel sheets were used for TLC analyses. GLC analyses were performed using two types of capillary columns: an Alltech AT-35 bonded FSOT column (30 m x 0.25 mm i.d.) and an Alltech AT-1 bonded FSOT column (30 m x 0.25 mm i.d.). Purifications by flash-chromatography were performed using silica gel Merck 60 (particle size 0.040–0.063 mm). EI-MS spectra were measured at 70 eV by GLC/MS. ESI-MS spectra were acquired on a PE Sciex API 365 coupled with a PerkinElmer 200 HPLC (Perkin Elmer, Wallac Oy, Turku, Finland), and were obtained under the following conditions: ionspray voltage, - 4.0 kV; orifice voltage, - 60 V; scan range, *m/z* = 400–600; scan time, 4.73 s; no interscan delay; unity-mass resolution. NMR spectra were recorded at room temperature at 200 and 500 MHz and were referred to TMS. All reactions were performed under argon, by standard syringe, cannula and septa techniques. *N*-phenylnaphthalen-2-amine, 7-amino-α-tetralone, bromobenzene, $Pd_2(dba)_3$, 2,2'-bis(diphenylphosphino)-1,1'-binaphthyl (BINAP), 10% Pd/C, were commercially available and were used as received. All the solvents were commercially available, and were used as received.

Sodium 6-(phenylamino)naphthalene-1-sulfonate (**SM.1-31**)

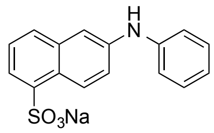

According to a literature procedure [1], *N*-phenylnaphthalen-2-amine (2.2 g, 10 mmol) was added at room temperature to 96% sulfuric acid (4.9 mL, 9 g). The resulting black solution was stirred for 5 h at 80 °C, then cooled to 0 °C and quenched with cold water. The grey solid obtained was collected by filtration and suspended in water. The mixture was heated up to the complete solubilization of the solid, and the resulting solution was neutralized with 2M aqueous NaOH. The neutral hot

solution was cooled to room temperature, and filtered to remove the solid that precipitated after cooling. The solution was evaporated to dryness and the solid obtained was finely triturated and extracted with boiling 96% ethanol. The alcoholic extract was concentrated at reduced pressure, and the solid so obtained was washed with hot toluene obtaining 0.44 g (14% yield) of **SM.1-31** as a white solid. The final compound was dissolved in 50 mM potassium phosphate buffer pH 5.5 (90% H_2O/10% D_2O) and characterized by ^1H and ^{13}C NMR. Spectra were recorded at 25°C on a 500 MHz DMX Bruker spectrometer. 2D ^1H-TOCSY (spin lock time 50 ms), ROESY (mixing time 250 ms), J-resolved and ^1H-^{13}C HSQC spectra, were used to assign compound resonances. The resonance assignment is reported in Suppl. Figure S3 and S4

Sodium 7-(phenylamino)naphthalene-1-sulfonate (**SM.1-32**)

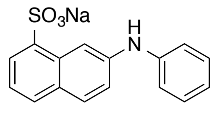

According to a literature procedure [1], *N*-phenylnaphthalen-2-amine (10 g, 45.6 mmol) was added at room temperature to 96% sulfuric acid (22.2 mL, 40 g). The resulting black solution was stirred for 5 h at 80 °C, then cooled to 0 °C and quenched with cold water. The solid obtained was collected by filtration and washed twice with water. The crude mixture of sulfonic acids **SM.1-31** e **SM.1-32** so obtained was recrystallized from EtOH/H_2O in order to separate the two isomeric acids. From the first crystallization the less soluble **SM.1-31** was recovered. The solid resulting from partial concentration of the mother liquor and subsequent recrystallization was dispersed in a small amount of EtOH/H_2O. The mixture was heated up to complete solubilization, and neutralized with 2M aqueous $NaOH_{aq}$, using phenolphthalein as indicator. The neutralized hot solution was filtered and cooled to RT, obtaining a solid. The white salt recrystallized was collected and was washed with a little amount of cooled water, obtaining 2.55 g (18% yield) of desired product. ESI-MS[M-Na]= 298. The ^1H NMR spectrum of the final compound, dissolved in 50 mM potassium phosphate buffer pH 5.5 (90% H_2O/10% D_2O) recorded at 25°C, is reported in Suppl. Figure S3.

8-Hydroxy-N-phenylnaphthalen-2-aminium chloride (**SM.1-33**)

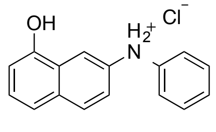

A mixture of 7-amino-α-tetralone (4.6 g 28.5 mmol), bromobenzene (2.5 mL, 3.74 g, 28.8 mmol,), $Pd_2(dba)_3$ (220 mg, 0.24 mmol), BINAP (224 mg, 0.36 mmol) and Cs_2CO_3 (10.85 g, 33.3 mmol) in 95 mL of dry toluene was stirred under Ar for 48 h at 100 °C. After cooling at room temperature, the reaction mixture was diluted with CH_2Cl_2, filtered through a short plug of celite and eluted with additional CH_2Cl_2. The filtrate was concentrated under reduced pressure and the residue was

purified by flash chromatography on silica gel (toluene/AcOEt : 95/5), affording 3.81 g (68% yield) of 7-(phenylamino)-3,4-dihydronaphthalen-1(2*H*)-one as a yellow solid. The chemical structure of the title compound was confirmed by NMR. ^{1}H-NMR (200 MHz) δ: 7.69 (1H, d, *J* = 2.4 Hz); 7.16 (7H, m); 5.80 (1H, bs); 2.89 (2H, t, *J* = 6 Hz); 2.64 (2H, d, *J* = 6.2 Hz); 2.11 ppm (2H, q, *J* = 6.2 Hz). ^{13}C-NMR δ: 198.6, 142.7, 141.8, 136.9, 129.6, 129.3 (2C), 123.1, 121.0, 117.6 (2C), 115.1, 39.1, 28.9, 23.4 ppm. MS (EI): m/z (%) 238 (18), 237 (100), 236 (36), 181 (40), 180 (39).

A mixture of of 7-(phenylamino)-3,4-dihydronaphthalen-1(2*H*)-one (3.16 g, 13.3 mmol) and 10% Pd/C (1.90 g, 13.3 mmol of Pd) in 330 mL of *p*-cymene was heated to reflux for 48 h. After cooling to room temperature, the reaction mixture was diluted with toluene, filtered through a short plug of celite and eluted with additional toluene and CH_2Cl_2. The solvents were removed in vacuo, and the resulting crude product was purified by flash chromatography on silica gel (petroleum ether/EtOAc: 80/20). The chromatographic fractions containing the product were collected and concentrated in vacuo. The orange solid so obtained was then dissolved in toluene, and gaseous HCl was bubbled into the solution. The newly formed solid was collected by filtration, repeatedly washed with toluene and hexane, dried in vacuo to give 8-hydroxy-*N*-phenylnaphthalen-2-aminium chloride (**SM.1-33**) as a white solid (2.33 g, 65 % yield). The ^{1}H NMR spectrum of SM.1-33, dissolved in 50 mM potassium phosphate buffer pH 5.5 (90% H_2O/10% D_2O) recorded at 25°C, is reported in Suppl. Figure S3.

Sodium 4-hydroxy-6-(phenylamino)naphthalene-1,3-disulfonate (**SM.1-34**).

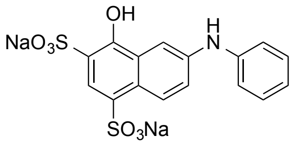

According to a literature procedure [1], 8-hydroxy-*N*-phenylnaphthalen-2-aminium chloride (0.5 g, 1.85 mmol) was added to 96% sulphuric acid (2 g, 1 mL). During the addition evolution of gas (HCl) was observed. The resulting mixture was heated at 80 °C for 5 h, and after cooling at 0 °C the reaction was quenched with water (4 mL). The mixture was warmed to room temperature, filtered, and the filtrate was neutralized with 2M aqueous NaOH. At pH 7 the formation of a pale green solid was observed. This solid was collected, and extracted with MeOH at room temperature. The alcoholic extracts were concentrated in vacuo, giving the required **SM.1-34** as a pale green solid (0,56 g, 70%). The ^{1}H NMR spectrum of SM.1-34, dissolved in 50 mM potassium phosphate buffer pH 5.5 (90% H_2O/10% D_2O) recorded at 25°C, is reported in Suppl. Figure S3.

Photon correlation spectroscopy and Zeta Potential analysis. Photon correlation spectroscopy (PCS) and the Zeta Potential (Zp) values were measured using the Zetasizer Nano ZS (Malvern, Worcestshire, UK) with a fixed 173° scattering angle and a 633-nm-helium-neon-laser. Data were analyzed by Zetasizer software, version 7.11 (Malvern, Worcestshire, UK). The set up temperature was 25°C.

Samples were dissolved in 1 ml of deionized water with 1% HBSS (Hanks' Balanced Salt solution) to obtain a concentration of about 3mg/ml. For PCS experiments the solution was further diluted with HBSS to the indicated concentration, transferred to a disposable folded capillary cell at room temperature and analyzed 10' minutes post-diluting.

For Zp measurements, 1mg/ml solution of the compounds were used. Due to the high conductivity of buffer solution precluding electrophoretic mobility measurements, about 3mg of samples were solubilized in 1% HBSS and diluted with suitable volume of deionized water. The solution, was transferred to a disposable folded capillary cell (Malvern, Worcestshire, UK) at room temperature and analyzed 10' post-diluting.

Reference

1. Green, A.G. & Vakil, K.H. VI. Studies on the sulphonylation of β-naphthylamine. *J Chem Soc* **113**, 35-44 (1918).

SUPPLEMENTARY TABLES

Suppl. Table S1. NSC number and IUPAC nomenclature of the inhibitors used in the study

Small molecule	**NSC**	**IUPAC**
SM27	NSC37204	6,6'-(carbonylbis(azanediyl))bis(4-hydroxynaphthalene-2-sulfonic acid)
SM.1-3	NSC1745	7-anilino-4-hydroxynaphthalene-2-sulfonic acid
SM.1-4	NSC2602	8-amino-4-hydroxynaphthalene-2-sulfonic acid
SM.1-5	NSC7560	3-amino-5-hydroxynaphthalene-2,7-disulfonic acid
SM.1-6	NSC7567	7-acetamido-4-hydroxynaphthalene-2-sulfonic acid
SM.1-7	NSC8631	7-amino-4-hydroxynaphthalene-2-sulfonic acid
SM.1-8	NSC8637	6-(dimethylamino)-4-hydroxynaphthalene-2-sulfonic acid
SM.1-9	NSC8660	6-anilino-4-hydroxynaphthalene-2-sulfonic acid
SM.1-13	NSC37190	7-(4-ethoxyanilino)-4-hydroxynaphthalene-2-sulfonic acid
SM.1-25	NSC134814	7-acetamido-4-hydroxynaphthalene-2-sulfonic acid
SM.1-31		sodium 6-(phenylamino)naphthalene-1-sulfonate
SM.1-32		sodium 7-(phenylamino)naphthalene-1-sulfonate
SM.1-34		sodium 4-hydroxy-6-(phenylamino)naphthalene-1,3-disulfonate
SM.1-33		8-hydroxy-N-phenylanaphthalen-2-aminium chloride
SM.2-1	NSC1698	7,7'-azanediylbis(4-hydroxynaphthalene-2-sulfonic acid)
SM.2-10	NSC12149	6,6'-((((sulfonylbis(4,1-phenylene))bis(azanediyl))bis(carbonyl))bis(azanediyl))bis(naphthalene-2-sulfonic acid)
SM.2-11	NSC12857	7,7'-(carbonylbis(azanediyl))bis(naphthalene-1,3-disulfonic acid)
SM.2-12	NSC16223	4-hydroxy-7-(3-(5-hydroxy-6-((*E*)-phenyldiazenyl)-7-sulfonaphthalen-2-yl)ureido)-3-((*E*)-phenyldiazenyl)naphthalene-2-sulfonic acid
SM.2-15	NSC45616	(*E*)-4-hydroxy-7-(3-(5-hydroxy-7-sulfonaphthalen-2-yl)ureido)-3-((6-sulfonaphthalen-2-yl)diazenyl)naphthalene-2-sulfonic acid
SM.2-16	NSC45622	3-((*E*)-(1-amino-6-sulfonaphthalen-2-yl)diazenyl)-4-hydroxy-7-(3-(5-hydroxy-6-((*E*)-(2-methoxyphenyl)diazenyl)-7-sulfonaphthalen-2-yl)ureido)naphthalene-2-sulfonic acid
SM.2-17	NSC47749	4-hydroxy-7-(3-(5-hydroxy-6-((*E*)-phenyldiazenyl)-7-sulfonaphthalen-2-yl)ureido)-3-((*E*)-phenyldiazenyl)naphthalene-2-sulfonic acid
SM.2-18	NSC47750	3-((*E*)-(3-aminophenyl)diazenyl)-7-(3-(6-((*E*)-(3-aminophenyl)diazenyl)-5-hydroxy-7-sulfonaphthalen-2-yl)ureido)-4-hydroxynaphthalene-2-sulfonic acid
SM.2-19	NSC47762	3-((*E*)-(4-acetamidophenyl)diazenyl)-4-hydroxy-7-(3-(5-hydroxy-6-((*E*)-phenyldiazenyl)-7-sulfonaphthalen-2-yl)ureido)naphthalene-2-sulfonic acid
SM.2-20	NSC58057	4-hydroxy-7-(3-(5-hydroxy-7-sulfo-6-((*E*)-(6-sulfonaphthalen-2-yl)diazenyl)naphthalen-2-yl)ureido)-3-((*E*)-*o*-tolyldiazenyl)naphthalene-2-sulfonic acid
SM.2-21	NSC65573	4-hydroxy-7-(3-(5-hydroxy-7-sulfo-6-((*E*)-(6-sulfonaphthalen-2-yl)diazenyl)naphthalen-2-yl)ureido)-3-((*E*)-*m*-tolyldiazenyl)naphthalene-2-sulfonic acid
SM.2-22	NSC65574	4-hydroxy-7-(3-(5-hydroxy-7-sulfo-6-((*E*)-(6-sulfonaphthalen-2-yl)diazenyl)naphthalen-2-yl)ureido)-3-((*E*)-*m*-tolyldiazenyl)naphthalene-2-sulfonic acid
SM.2-23	NSC65575	3-((*E*)-(2,4-dimethylphenyl)diazenyl)-4-hydroxy-7-(3-(5-hydroxy-7-sulfo-6-((*E*)-(6-sulfonaphthalen-2-yl)diazenyl)naphthalen-2-yl)ureido)naphthalene-2-sulfonic acid
SM.2-24	NSC65576	3-((*E*)-(2,5-dimethylphenyl)diazenyl)-4-hydroxy-7-(3-(5-hydroxy-7-sulfo-6-((*E*)-(6-sulfonaphthalen-2-yl)diazenyl)naphthalen-2-yl)ureido)naphthalene-2-sulfonic acid

Suppl. Table S2. Determination of the affinity (K_d) of the FGF2/compound interactions by SPR analysis.

Compound	K_d	X^2
SM27	335	0.7
SM.2-16	125	2.5
SM.2-20	318	1.3
SM.2-22	39	1.3
SM.2-23	93	1.1
SM.2-24	111	1.8

Binding parameters were calculated by the non-linear curve fitting software package BIAevaluation 3.2 using a single site model with correction for mass transfer. Dissociation constant (K_d) values were derived from the dissociation rate/association ratio. Only sensorgrams whose fitting gave values of X^2 lower than 10 were used {Khalifa, 2001 #213}. The data shown are from one experiment representative of two that gave similar results.

SUPPLEMENTARY FIGURES

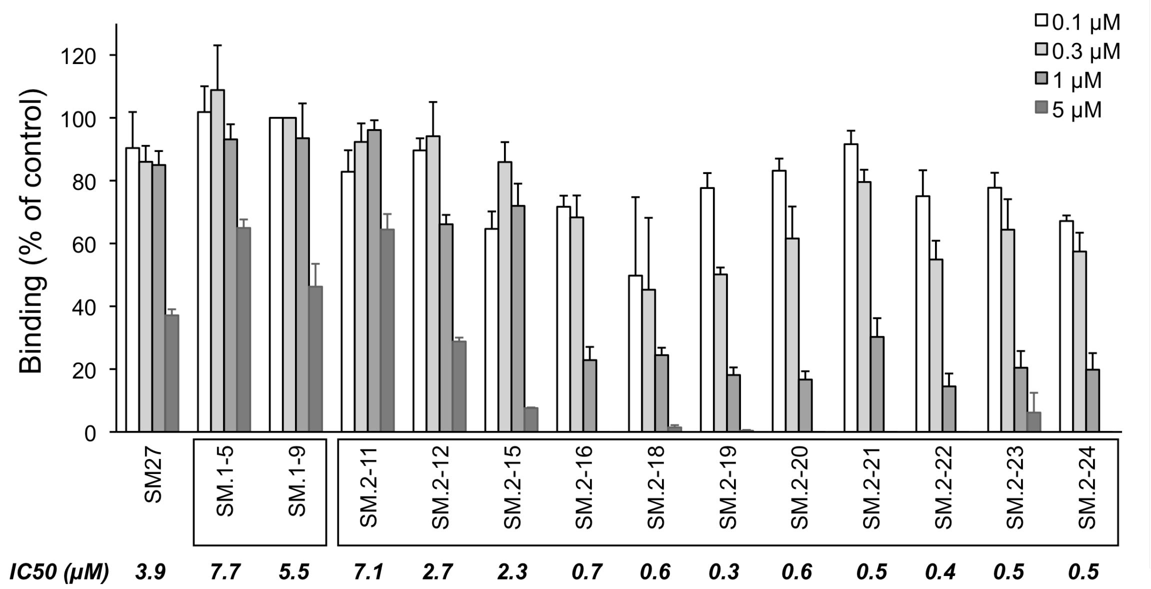

Suppl. Figure S1

Dose-dependent inhibition of the binding of the TSP-1 domain to FGF2 by the new hits. The assay was conducted as in Figure 2, testing each molecule at the indicated concentration. Data are the percentage of control binding domain, average and standard error of 3 experiments performed in triplicate.

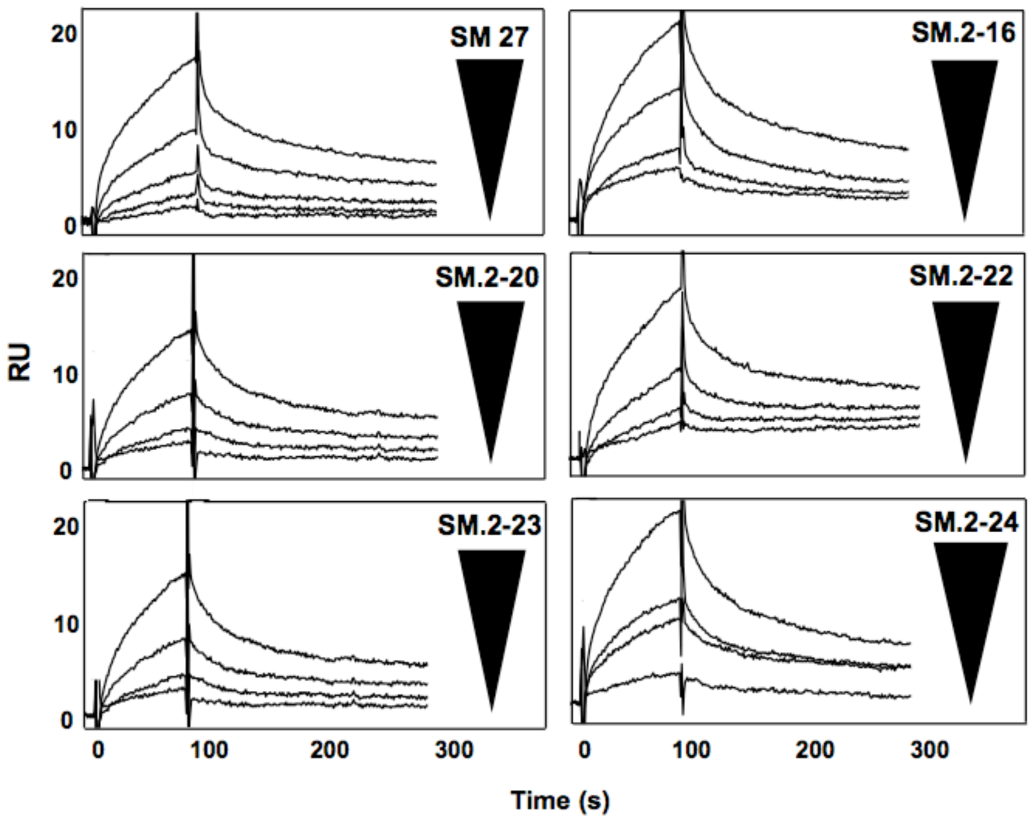

Suppl. Figure S2

Representative SPR sensorgram overlays of the binding of increasing concentrations of the indicated hit to sensorchip-immobilized FGF2.

SM.1-31

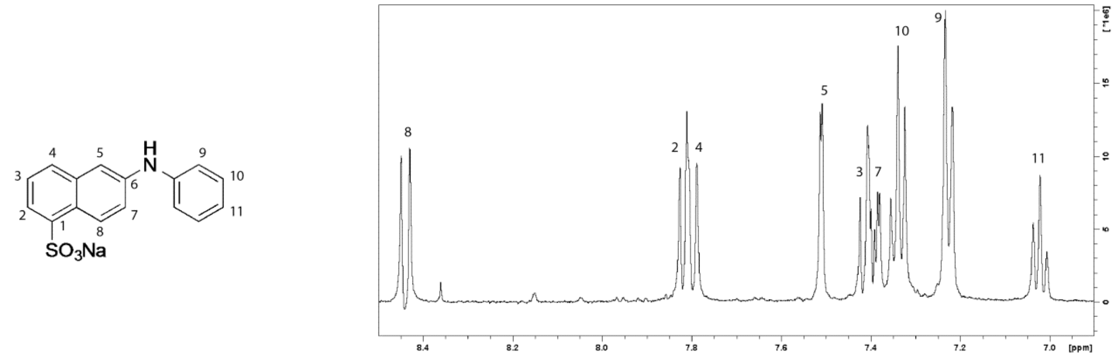

SM.1-32

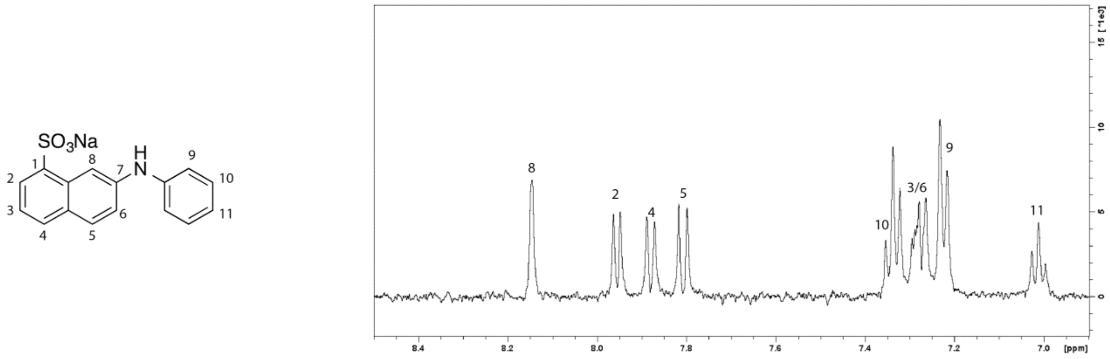

SM.1-33

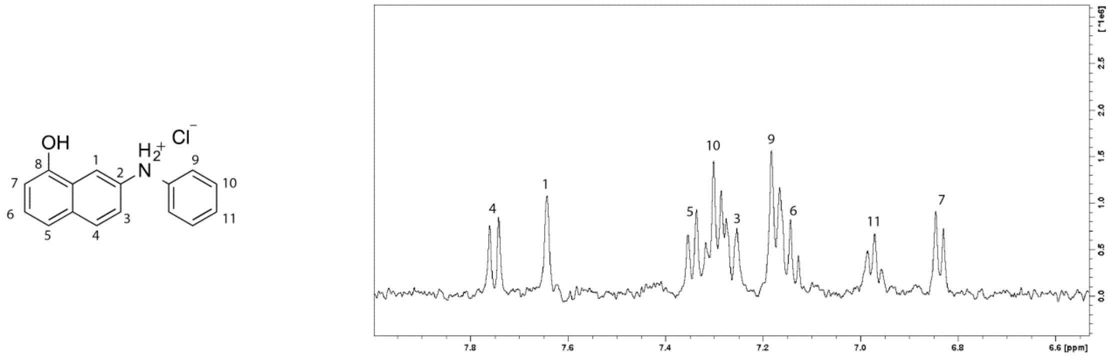

SM.1-34

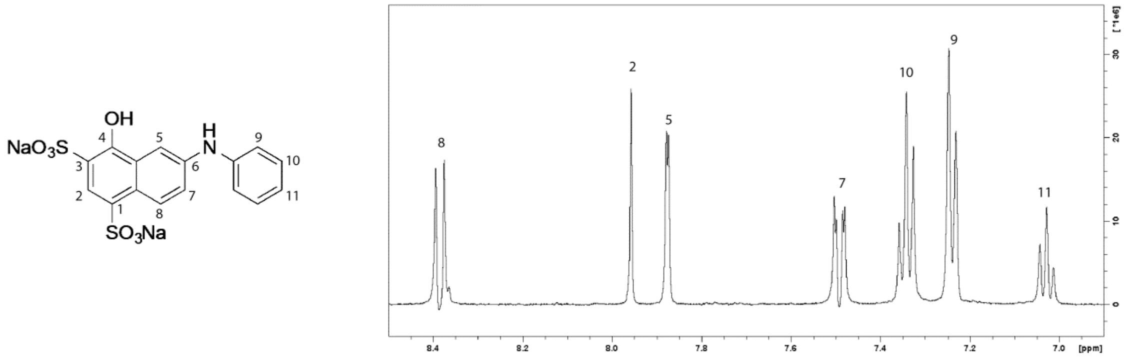

Suppl. Figure S3

1D ^{1}H spectrum of SM.1-31, SM.1-32, SM.1-33, and SM.1-34 in 50mM potassium phosphate buffer pH 5.5, 25 °C, recorded on a Bruker 500 MHz spectrometer. Resonance assignment is reported.

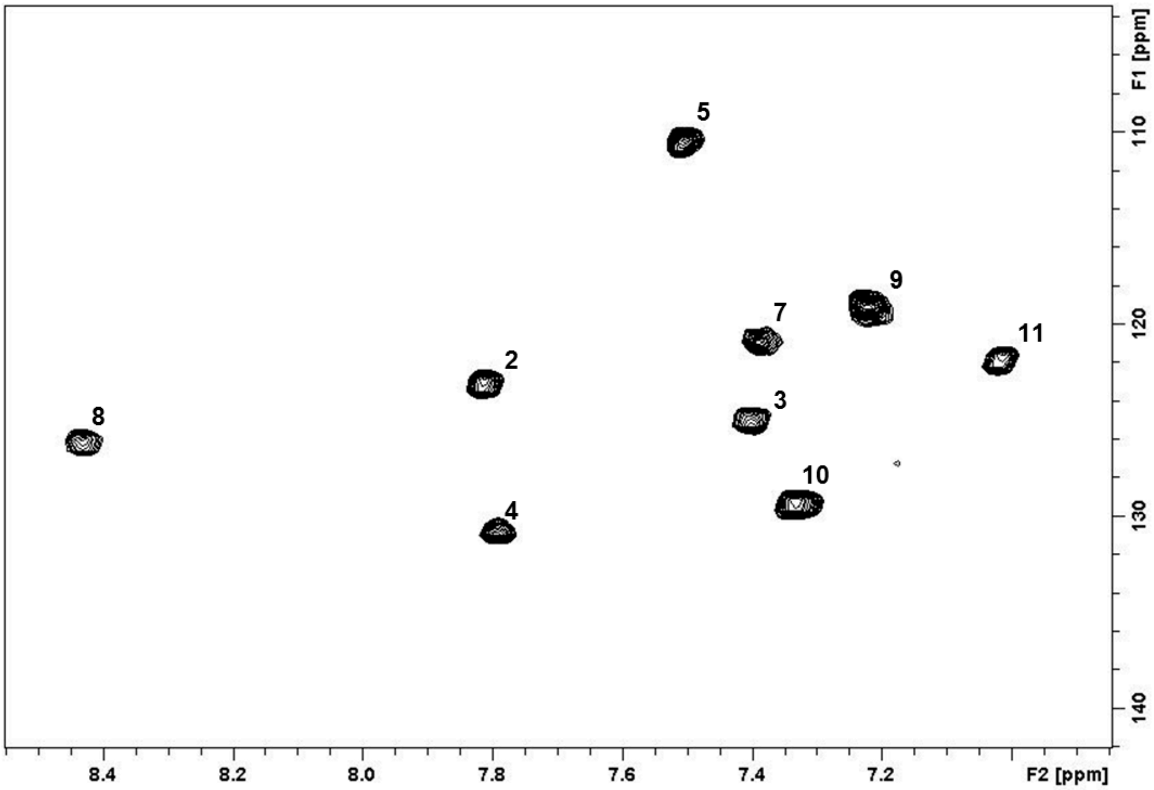

Suppl. Figure S4.

2D $^{1}H^{13}C$-HSQC spectrum of SM.1-31 in 50mM potassium phosphate buffer pH 5.5, 25 °C, recorded on a Bruker 500 MHz spectrometer. Resonance assignment is reported. Atom numbering as in Suppl. Figure S3
